# Systematic interrogation of the *Conus marmoreus* venom duct transcriptome with ConoSorter reveals 158 novel conotoxins and 13 new gene superfamilies

**DOI:** 10.1186/1471-2164-14-708

**Published:** 2013-10-16

**Authors:** Vincent Lavergne, Sébastien Dutertre, Ai-hua Jin, Richard J Lewis, Ryan J Taft, Paul F Alewood

**Affiliations:** 1 Division of Chemistry and Structural Biology, Institute for Molecular Bioscience, The University of Queensland, Brisbane, Qld 4072, Australia; 2 Division of Genomics and Computational Biology, Institute for Molecular Bioscience, The University of Queensland, Brisbane, Qld 4072, Australia

**Keywords:** ConoSorter, Cone snail, Venomics, Transcriptome, Proteome, Conopeptides, Conotoxins

## Abstract

**Background:**

Conopeptides, often generically referred to as conotoxins, are small neurotoxins found in the venom of predatory marine cone snails. These molecules are highly stable and are able to efficiently and selectively interact with a wide variety of heterologous receptors and channels, making them valuable pharmacological probes and potential drug leads. Recent advances in next-generation RNA sequencing and high-throughput proteomics have led to the generation of large data sets that require purpose-built and dedicated bioinformatics tools for efficient data mining.

**Results:**

Here we describe ConoSorter, an algorithm that categorizes cDNA or protein sequences into conopeptide superfamilies and classes based on their signal, pro- and mature region sequence composition. ConoSorter also catalogues key sequence characteristics (including relative sequence frequency, length, number of cysteines, N-terminal hydrophobicity, sequence similarity score) and automatically searches the ConoServer database for known precursor sequences, facilitating identification of known and novel conopeptides. When applied to ConoServer and UniProtKB/Swiss-Prot databases, ConoSorter is able to recognize 100% of known conotoxin superfamilies and classes with a minimum species specificity of 99%. As a proof of concept, we performed a reanalysis of *Conus marmoreus* venom duct transcriptome and (i) correctly classified all sequences previously annotated, (ii) identified 158 novel precursor conopeptide transcripts, 106 of which were confirmed by protein mass spectrometry, and (iii) identified another 13 novel conotoxin gene superfamilies.

**Conclusions:**

Taken together, these findings indicate that ConoSorter is not only capable of robust classification of known conopeptides from large RNA data sets, but can also facilitate *de novo* identification of conopeptides which may have pharmaceutical importance.

## Background

Venomous marine cone snails have evolved a broad array of peptide toxins, called conopeptides, for prey capture and defense. These small bioactive compounds selectively act on a wide variety of receptors and channels in the central and peripheral nervous systems [1-4]. These vast, mostly untapped, natural toxin libraries provide potent tools for studying the properties of these targets and have become a platform for the discovery of new pharmaceuticals [5-8]. Only ~2% of the estimated >70,000 venom peptides expressed by the genus *Conus* have been sequenced to date [9].

**Figure 1:**
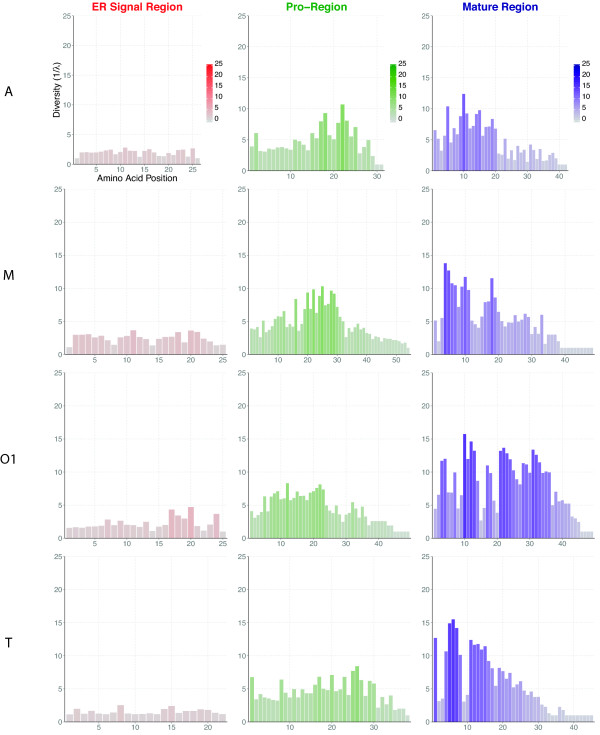
**Amino acid diversity in conopeptides.** The position-specific diversity of amino acid for each conopeptide regions (ER signal in red, pro- in green, and mature region in purple) belonging to the 4 largest gene superfamilies A, M, O1 and T (the remaining superfamilies are presented in Additional file 1: Figure S1). The true diversity of order 2 (or inverse Simpson index, 1/λ) have been calculated according to the following equation in order to take into account the amino acid richness R, their average proportional abundance p_i_, as well as the variability of sequence lengths for each regions: $1/\lambda =1/\sum_{i=1}^{R}p_{i}^{2}$. For each amino acid position, a color gradient applies to the diversity index (from 0 in light to higher values in darker color).

In the apical secretory cells lining the long convoluted venom duct [10,11] (and likely to a much lesser extent the salivary glands [12]), mature mRNA is translated to precursor conopeptides which are generally composed of three distinct regions: a N-terminal endoplasmic reticulum (ER) signal sequence, a central pro-peptide region, and the C-terminal mature toxin. Based on the conservation of their signal sequence, conopeptides are currently classified into 16 empirical gene superfamilies (A, D, I1, I2, I3, J, L, M, O1, O2, O3, P, S, T, V, Y), and 13 minor families for those identified in early divergent clade species [13-16]. In addition, 10 new superfamilies have been discovered in the past two years - B1 [17], B2 [18], B3 [19], C [17], E [18], F [18], G [20], H [18], K [21], N [18]. Conopeptides can also be further divided into secondary classes based on the number of disulfide bonds they can contain - disulfide-rich conopeptides containing at least 2 disulfide bonds are colloquially known as conotoxins, whereas those with none or one disulfide bond are called disulfide-poor conopeptides [22] - or the cysteine patterns in the mature region of disulfide-rich conopeptides [14]. Although amino acid conservation in the pro- and mature regions of conopeptides from the same superfamily is much lower than for the ER signal sequence (Figure 1 and Additional file 1: Figure S1), consensus cysteine patterns and connectivities are often highly conserved (although not always specific to a gene superfamily) and may be linked to particular pharmacological families [14].

Recent studies have reported the existence of new conopeptides, which do not clearly belong to any of the previous annotated superfamilies but share common pharmacological targets. Although some show conserved signal regions, cysteine motifs or specific post-translational modifications, these conotoxins have been incorporated into 14 additional classes [14] called conantokin [23], conodipine [24], conohyal [25], conolysin [26], conomap [27], conomarphin [28], conopeptide Y [29], conophan [30], conoporin [31], conopressin [32], conorfamide [33], conotoxin-like [12], contryphan [34] and contulakin [35].

Advances in high-throughput sequencing technologies, combined with directed studies of venom producing cells [36-39], have resulted in a data deluge which requires dedicated tools for the analysis and classification of conopeptide sequences. ConoServer, a specialized database dedicated to conopeptides [22], implemented a web-based tool (*ConoPrec*) that guides gene superfamily assignment of precursor toxins by the recognition of a limited number of known cleavage sites (or protease specificities) and a sequence similarity search based on existing conopeptide superfamilies [16]. However, the limitations of this program include the restriction to known conopeptide motifs, as well as a requirement that the query precursor sequences contain the signal region, which is rarely the case as most conopeptide screening is conducted on milked venom or dissected venom gland that almost exclusively contains mature protein products. Another web-based program, *ConoDictor*, overcomes the issue of missing signal regions by using three independent sets of models built from signal, pro- and mature regions of conopeptides respectively [40,41]. However, this tool only accepts selected amino acid sequences as input, only classifies conopeptides into the main superfamilies, does not provide any data quantitation, and perhaps most importantly, cannot facilitate the discovery of new conopeptide families. Both *ConoPrec* and *ConoDictor* are limited in their ability to handle large transcriptomic or proteomic datasets, and therefore are unlikely to fill the need for large-scale analysis of cone snail transcriptomes or proteomes.

Here we describe ConoSorter, a program able to classify conopeptides into superfamilies and classes from either protein sequences or RNA sequencing data. ConoSorter has been designed to recognize all currently annotated gene superfamilies and classes. Regular expression sequence searches are complemented by a profile Hidden Markov Model (pHMM) analysis allowing the classification of conotoxins that may be only distantly related to well-established conopeptide groups. ConoSorter also reports key sequence characteristics (including relative sequence frequency, length, number of cysteine residues, N-terminal hydrophobicity, sequence similarity score) and automatically searches the ConoServer database for known precursor sequences, which facilitates clear and precise identification of known and novel conopeptides and their associated families. ConoSorter allows an investigator to efficiently deal with the thousands of sequences produced by high-throughput sequencing methods in a rapid and accurate manner.

## Results

### Identification and classification of known conopeptides

To assess if ConoSorter can accurately classify conopeptides into superfamilies and classes we performed two initial control experiments - analysis of the ConoServer cone snail toxin database and an analysis of the universal UniProtKB/Swiss-Prot protein database [22,42].

The ConoServer database contains 5,449 entries of complete or partial conopeptide sequences. We employed 36.85% (2,008 sequences) of the ConoServer entries in the development of our training set, and here sought to assess the accuracy of both the regular expression and pHMM approaches described above to hierarchically classify the entire suite of ConoServer sequences into superfamily and class. We found that the regular expression analysis was able to classify 100% of well-defined ConoServer sequence regions (i.e. those that do not display undetermined amino acids) for which the gene superfamily or class have been previously assigned. This approach also assigned a superfamily to 1,228 sequences, and a class to 42 others, which were not previously classified. ConoSorter failed to confidently classify a total of only ~440 sequences, all of which are derived from patents and synthetic constructs that contain one or more undetermined amino acids, or are sequences for which supportive data regarding their classification are unavailable or have been predicted with an unknown level of accuracy.

Analysis of the ConoServer entries with pHMMs showed true positive recognition rates of 99.25% for superfamily (9 instances of conopeptide region annotation conflict, plus 3 false positives out of 1,609 complete sequences with annotated superfamilies), and 99.60% for class (7 conflicts, plus 4 patent sequences with undetermined amino acids counted as false positives out of 2,750 sequences with known classes) using the HMMER *hmmscan* script with the default E-value cutoff at 10. This approach was also able to confidently assign 1,153 sequences into superfamilies and 32 into classes, which had previously lacked annotation.

In the second experiment, the ability of ConoSorter to distinguish between *Conus* peptide toxins and other proteins from various organisms has been assessed by screening the entire UniProtKB/Swiss-Prot database. Using the version released on June 2013 we examined a total of 540,261 protein sequences isolated from 12,988 cellular and non-cellular species. Table 1 reports the specificity *S* calculated at 7 E-value cutoffs (10 – default threshold, 1, 0.1, 0.01, 10^-3^, 10^-4^ and 10^-5^) according to the following equation:

$$S=\frac{\text{True}\;\text{Negatives}}{\left(\text{True}\;\text{Negatives}+\text{False}\;\text{Positives}\right)}$$

where *True Negatives* = *N* – *True Positives* (with *N* = total number of input sequences, and *True Positives* = number of conopeptide matches), and *False Positives* = the number of non-*Conus* species matches plus the number of non-conopeptides *Conus* matches.

**Table 1:** Species specificity of conopeptide models

***E-value cutoff***	***Superfamily***		***Class***	
	***S (%)***	***False + (%)***	***S (%)***	***False + (%)***
≤10	99.19	0.81	99.25	0.75
≤1	99.31	0.69	99.35	0.66
≤0.1	99.42	0.58	99.46	0.55
≤0.01	99.56	0.44	99.57	0.43
≤10^-3^	99.81	0.19	99.81	0.19
≤10^-4^	99.90	0.10	99.90	0.10
≤10^-5^	99.94	0.06	99.93	0.07

The species specificity, S (expressed in %), of the conopeptide models has been assessed on UniProtKB/Swiss-Prot database (540,261 sequences in total spread over 12,988 species) at different total E-value thresholds. Percentages of specificity and false positive rates (“False +”, expressed in %) are reported for the classification by gene superfamily, and by class.

At all E-value thresholds ConoSorter was able to confidently identify and classify conopeptides (Table 1). Of the 540,261 amino acid sequences referenced in the UniProtKB/Swiss-Prot database, ConoSorter, using an E-value of 10^-5^, was able to classify 879 peptide toxins from the genus *Conus* with an annotated superfamily (specificity of 99.94%) with only 345 false positives (p-value = 0.06%) isolated from other organisms. Similarly, ConoSorter was able to classify 894 conopeptides with an annotated class (specificity of 99.93%) with only 393 false positives (p-value = 0.07%).

### Analysis of *Conus marmoreus* venom duct transcriptome

The results presented above indicate that ConoSorter is capable of identifying conopeptides at high specificity and sensitivity, and, even when the dataset being analyzed ostensibly includes all known proteins, accurately assigning the appropriate superfamily and class. We next sought to use ConoSorter’s regular expression and pHMMs searches to ascertain if it was possible to identify novel conopeptides, superfamilies and classes in a previously interrogated RNA-seq dataset.

Dutertre *et al.* have recently performed an analysis of the *C. marmoreus* venom duct transcriptome, which principally relied on serial BLAST homology searches [18]. They reported 30 full conopeptides precursor sequences (i.e. those beginning with a methionine residue and finishing by a stop codon) that had been previously characterized in this species. A total of 75 novel conopeptides were also identified which were assigned to 8 known gene superfamilies. Thirteen of these were classified, based on the high conservation of their signal sequences, into 5 new superfamilies dubbed B2, E, F, H, and N.

We re-examined this medium-throughput 454 sequencing data (179,843 cDNA sequences) with ConoSorter, and identified 4,307,681 putative precursor protein sequences derived from all possible translations of these sequences into six reading frames (see *ConoSorter pipeline* in Methods), which were analyzed hierarchically using the regular expressions and pHMMs described above to assign sequences to superfamily, class or 'unknown’. This led to the identification of 72% (106/146) of annotated complete *Conus marmoreus* precursor conopeptides, including all but two of the sequences found in the previous manual analysis (two incomplete conotoxins, named Mr8.1 and Mr11.3 precursors, did not encode a methionine and thus were discarded). Moreover, 17 novel isoforms of known *Conus marmoreus* precursor conopeptides (Mr1.1, conomarphin Mr1, conomarphin Mr2, contryphan M, cMrVIA, CMrX, and MrIA precursors) were identified and assigned the correct superfamily or class based on the signal, pro-, and mature regions. These conopeptides were confirmed by a tandem mass spectrometry (MS/MS) analysis of the milked venom, in which we were able to identify their corresponding mature sequences (Additional file 2: Table S1).

**Figure 2:**
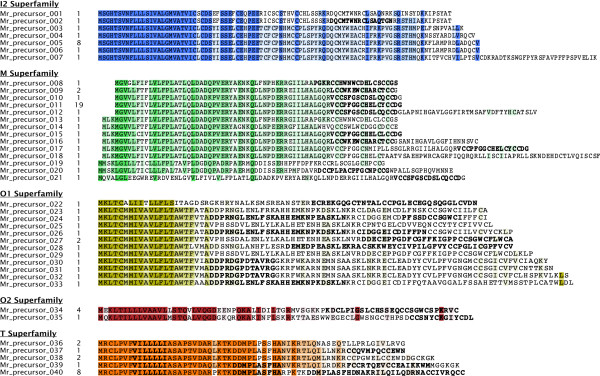
**New conopeptide precursors isolated from the venom duct transcriptome of *Conus marmoreus *and MS/MS coverage.** New full-length precursor conopeptides inferred from mRNA data, and detected with ConoSorter simultaneously in the signal, pro-, and mature regions without conflict (isoforms of these sequences can be found in Additional file 3: Figure S2). For each known superfamily, the sequence alignment shows the amino acid conservation specifically in the N-terminal signal region (the deeper color the more conserved the amino acids are). Partial sequences in bold characters correspond to the peptide fragments isolated by MS/MS analysis of *C. marmoreus* milked venom. Numbers listed after conopeptide names indicate the sequence frequency in the input data. DNA Data Bank of Japan (DDBJ) accession numbers of full-length precursors, as well as post-translational modifications (PTMs) calculated by *ProteinPilot 4.0* are provided in Additional file 4: Table S2.

ConoSorter was also able to assign known gene superfamilies to 125 novel full-length precursor conopeptides (Figure 2, Additional file 3: Figure S2, Additional file 4: Table S2). Using the *C. marmoreus* milked venom mass spectrometry data, and employing the methods Dutertre *et al.* used to match the MS data to putative novel conopeptides (e.g. restricting *ProteinPilot* results to those with a confidence threshold of at least 99%) [18], we were able to validate protein fragments of 86 of the novel conopeptide precursors (Figure 2, Additional file 3: Figure S2, Additional file 4: Table S2). Milked venom almost exclusively contains mature peptide toxins, which was reflected in the coverage of the mature peptide fragments compared to the full-length precursor conopeptide sequences (Figure 2).

**Figure 3:**
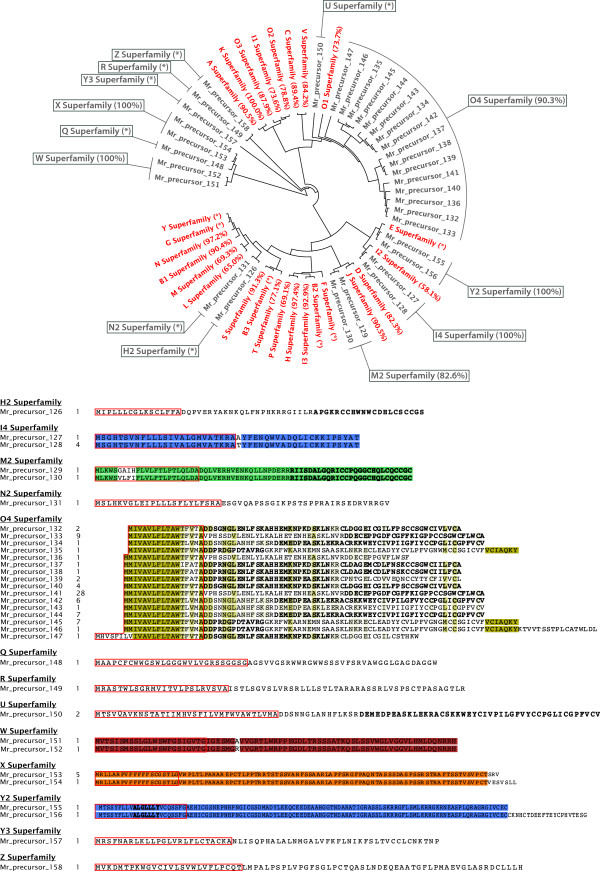
**New superfamilies of conopeptides.** Top panel shows an identity cladogram in which signal sequences of conopeptides matching only pro- and mature regions (in grey) have been aligned with consensus signal regions of known superfamilies (in red). Percentage value between brackets following the superfamily name measures the intraspecific conservation of members populating this family (the “*” symbol means that only one sequence is part of the family). Bottom panel shows the 33 new precursor sequences spread over the 13 new superfamilies. Red rectangles enclose the signal sequences determined with SignalP 4.0. The number following the precursor name is the precursor sequence frequency among the input data set. Bold partial sequences represent the peptide fragments retrieved by MS/MS sequencing of milked venom.

ConoSorter also identified 33 additional precursor conopeptides which, despite showing conserved amino acids and high hydrophobicity in the signal region, could not be classified into known superfamilies (Figure 3, Additional file 4: Table S2, Additional file 5: Table S3). Among these new precursors, 20 peptide fragments were identified in milked venom MS data (validation rate of ~60%). Based on their conservation, and their similarities with known superfamilies, we propose classifying these 33 precursor conopeptides into 13 new gene superfamilies - H2, I4, M2, N2, O4, Q, R, U, W, X, Y2, Y3, and Z. The names of these new groups have been taken from (i) the 6 available letters of the alphabet used to name the currently known superfamilies – Q, R, U, W, X, Z, or (ii) the names of the superfamily which they are the most similar to, and from which a number has been appended. For example, H2 is 40% similar to superfamily H (Figure 3, Additional file 5: Table S3). The signal sequences of these 13 new groups of conopeptides show high intrinsic identity rates from 82.6% to 100% (as a comparison, the lowest intraspecific conservation percentages known are 58.1%, 65.0% or 69.1% between members of the well established I2, L and P superfamilies). In addition, we find that the integration of these new distinct and well-defined superfamilies in the current classification does not interfere with the established superfamily classifications - for example, identity rates are as low as 0.2% between the new W group and the empirical I3 superfamily (Additional file 5: Table S3). ConoSorter also assigned non-conotoxin classes to 4 conomarphin and 2 contryphan precursor sequences (data not shown).

## Discussion

Many studies have reported the existence of intraspecific variations in the venom content of distinct *Conus* individuals belonging to the same species [43-46]. Reanalysis of the venom gland transcriptome of one *Conus marmoreus* individual revealed that ConoSorter was able to identify 72% of the annotated and complete known precursor conopeptides previously isolated in this species, and also led to the discovery of 158 new precursor conopeptides, 67.1% of which were validated in a matched MS/MS dataset. Interestingly, we observed that the overall number of *C. marmoreus* precursor conopeptides found to date is comparable to the number *Conus* species can theoretically produce [9]. Further investigation of the novel sequences identified by ConoSorter also allowed us to define 13 new superfamilies of conopeptides, which we have classified as H2, I4, M2, N2, O4, Q, R, U, W, X, Y2, Y3, and Z based on their intraspecific conservation rates and identity to established superfamilies. We note that in all new precursor sequences we were able to detect putative pro-peptide cleavage sites (usually positively charged amino acids like KR, LR or QR for instance), located just before the mature regions, an observation that is consistent with mass spectrometry data and supports the reliability of the matching between the venom duct transcriptome and the proteome of milked venom.

In this reanalysis we were able to retrieve 106 of the 146 known *Conus marmoreus* precursor sequences. A manual investigation of the 40 conopeptide sequences ConoSorter failed to identify in this analysis and the 454 RNA-seq data, revealed two likely sources of error: (i) 454 sequencing errors, particularly those associated with homopolymers (which has been extensively documented [47]), and (ii) lack of congruence between the RNA-seq data read length and the length of the encoded conopeptides. Indeed, although conopeptide precursors are relatively short polypeptides, their average length is nonetheless ~70 amino acids (~210 nucleotides), there are those, including CalMKLL-1 and -2 conotoxin precursors from *Conus californicus*, that are 131 amino acids in length. The average length of a high quality RNA-seq read in this dataset was 317.93 bases, indicating that failure to detect known conotoxins could be improved with longer reads. We suspect that further work in this field will take advantage of platforms offering up to 2 × 300 bp nucleotide reads, which not only allow for improved detection of conotoxins but may also facilitate de novo assembly of the *Conus* transcriptome.

## Conclusions

In this article we present ConoSorter, a high-throughput standalone program that implements regular expressions and pHMMs for large-scale identification and classification of precursor conopeptides into gene superfamilies and classes based on the ER signal, pro-, and mature conopeptide regions generated from raw next-generation transcriptomic or proteomic data. ConoSorter also generates a set of relevant additional information - frequency of protein sequences, length, number of cysteine residues, hydrophobicity rate of N-terminal region, similarity to known conopeptides - that allows the user to assess the reliability and relevance of the results and aids the identification of new conopeptide superfamilies and classes.

When applied to ConoServer and UniProtKB/Swiss-Prot databases, ConoSorter is able to recognize 100% of known conotoxin superfamilies and classes with a minimum species specificity of 99%. We also performed a reanalysis of *Conus marmoreus* venom duct transcriptome and (i) correctly classified all sequences previously annotated, (ii) retrieved 106 of the 146 precursor conopeptides known in this species, (iii) assigned the correct classification to 17 novel precursor toxin isoforms, (iv) identified 158 novel precursor conopeptide transcripts, 106 of which were confirmed by protein mass spectrometry, and (v) identified another 13 novel conotoxin gene superfamilies called here H2, I4, M2, N2, O4, Q, R, U, W, X, Y2, Y3, and Z.

Overall, ConoSorter provides a fully automated, accurate and easy-to-use tool for the analysis of large quantities of transcriptomic or proteomic data of conopeptide sequences, which could contribute to the acceleration of the discovery of new bioactive molecules.

## Methods

### Training set

Query data are compared to known conopeptide sequences using regular expressions and pHMMs. The conopeptides sequences used to build these reference models were obtained from the latest updates of the ConoServer (27/05/2013) and UniProtKB (06/2013) databases. These databases contain complete or partial wild type precursor and mature toxin sequences, isolated either as conotoxin genes, transcripts, or proteins, as well as artificially synthesized peptides. For the training set, only full-length endogenous precursor and mature conopeptides isolated at the protein level, or sequences from a superfamily where all members were annotated at the gene/DNA level, were used. Synthetic constructs and patented sequences with undetermined amino acids have not been included in the training set. A total of 2,008 sequences were used – 1,390 conopeptide superfamily sequences and 1,931 for their classes (a high proportion of which overlap). For each superfamily and class the signal, pro- and mature regions, which are used for the region-specific queries described below, were identified (Table 2). A total of 1,435 signal, 2,391 pro-, and 3,187 mature fragments were retrieved after discarding sequences with undetermined amino acids and duplicate regions coming from both the precursor and mature forms of the same conopeptide.

**Table 2:** Training set used to build regular expression and pHMMs models

	***Signal***			***Pro-region***			***Mature***		
	**Total clusters**	**Unique seq.**	**Total seq.**	**Total clusters**	**Unique seq.**	**Total seq.**	**Total clusters**	**Unique seq.**	**Total seq.**
***A***	3	-	77	21	6	136	23	6	195
***B1***	2	1	8	2	-	13	7	3	13
***B2***	1	1	1	1	1	1	1	1	1
***B3***	1	1	1	-	-	-	1	1	1
***C***	1	-	3	2	-	4	2	-	4
***D***	1	-	10	3	1	21	5	1	30
***E***	1	1	1	-	-	-	1	1	1
***F***	1	1	1	1	1	1	1	1	1
***G***	1	1	1	1	1	1	1	1	1
***H***	1	-	2	3	2	5	4	2	7
***I1***	3	1	12	5	4	13	8	2	48
***I2***	2	-	31	14	6	32	12	5	50
***I3***	1	-	4	3	2	7	2	-	8
***J***	1	-	8	2	1	5	2	-	11
***K***	1	1	1	1	1	1	1	-	3
***L***	4	2	10	5	2	10	5	2	11
***M***	6	2	152	16	5	263	34	16	267
***N***	1	-	2	1	-	3	1	-	3
***O1***	14	3	198	20	7	292	32	4	437
***O2***	4	1	50	15	6	75	12	6	81
***O3***	1	-	16	7	5	21	3	1	26
***P***	2	1	6	5	3	7	3	1	12
***S***	4	3	7	3	-	9	5	1	14
***T***	3	-	79	18	10	112	12	6	138
***V***	1	-	2	1	-	2	1	-	2
***Y***	1	1	1	1	1	1	1	1	1
***M---L-LTVA***	1	-	5	2	1	8	4	2	8
***MKFPLLFISL***	1	1	1	1	1	1	1	1	1
***MKLCVVIVLL***	1	-	2	1	1	1	1	-	3
***MKLLLTLLLG***	1	1	1	-	-	-	-	-	-
***MKVAVVLLVS***	1	1	1	-	-	-	-	-	-
***MRCLSIFVLL***	1	-	2	1	1	1	1	1	1
***MRFLHFLIVA***	1	1	1	1	1	1	1	1	1
***MRFYIGLMAA***	1	-	2	1	-	3	1	-	4
***MSKLVILAVL***	1	1	1	1	1	1	1	1	1
***MSTLGMTLL-***	1	-	5	3	2	5	3	1	5
***MTAKATLLVL***	1	1	1	1	1	1	1	1	1
***MTFLLLLVSV***	1	1	1	1	1	1	1	1	1
***MTLTFLLVVA***	1	1	1	1	1	1	1	1	1
***Conantokin***	2	1	8	2	-	13	4	1	19
***Conodipine***	-	-	-	-	-	-	2	2	2
***Conohyal***	1	-	2	-	-	-	2	2	2
***Conolysin***	-	-	-	-	-	-	1	-	2
***Conomap***	-	-	-	-	-	-	1	1	1
***Conomarphin***	1	-	2	1	-	2	1	-	4
***Conopeptide Y***	1	1	1	1	1	1	1	-	2
***Conophan***	-	-	-	-	-	-	1	-	2
***Conoporin***	1	1	1	-	-	-	1	1	1
***Conopressin***	1	1	1	-	-	-	1	-	6
***Conorfamide***	-	-	-	-	-	-	1	-	2
***Conotoxin***	29	4	697	84	14	1,299	189	87	1,730
***Conotoxin-like***	1	-	2	1	1	1	1	-	2
***Contryphan***	2	1	10	1	-	13	2	1	15
***Contulakin***	1	-	3	1	-	3	2	1	4

For each gene superfamilies and classes the table shows the total number of clusters (containing conopeptides with high sequence similarities), unique sequences, and total sequences in the ER signal, pro- and mature regions.

Each conopeptide region was aligned using ClustalW (BLOSUM cost matrix; gap open and extension penalties of 10 and 0.1 respectively) in order to generate clusters of closely related sequences and establish consensus subsets that best describe each superfamily and class. Signal conopeptide regions showed high conservation rates (from 61.4% to 100% identity). Sequence variability was higher in pro-region groups, and *CD-hit*[48], *MEME*[49], or *BLASTCLUST*[50] failed to produce reliable sequence clusters. Sequences were therefore manually curated until at least 40% pairwise identity was reached. Mature region sequence clusters were initially generated by analysis of the cysteine frameworks using a previously published in-house algorithm [51], which resulted in overall sequence conservation comparable to pro-regions. These clusters of sequences sorted by (i) superfamily, (ii) class, (iii) conopeptide signal, pro-, and mature regions and (iv) similarity were then used as templates for the creation of regular expressions. We produced 436 distinct models for superfamily classification (75, 165 and 196 for signal, pro-, and mature regions respectively) and 341 class models (40, 91, and 210 for signal, pro, and mature regions respectively). The number of clusters containing sequences with the highest rate of similarity and/or groups with unique sequence for each superfamily and class are summarized in Table 2.

The second sequence analysis approach implemented by ConoSorter is based on pHMMs. The sequence clusters described above used to create the regular expressions for each conopeptide superfamily / class have been used to build these models. Aligned clusters of sequences were converted to Stockholm format and the pHMMs were generated with *hmmbuild* from HMMER 3.0 package [52-54]. These conopeptide-specific profiles have then been concatenated to one single HMM database flat file, which has been subsequently compressed and indexed by using *hmmpress* from the same package.

### ConoSorter pipeline

**Figure 4:**
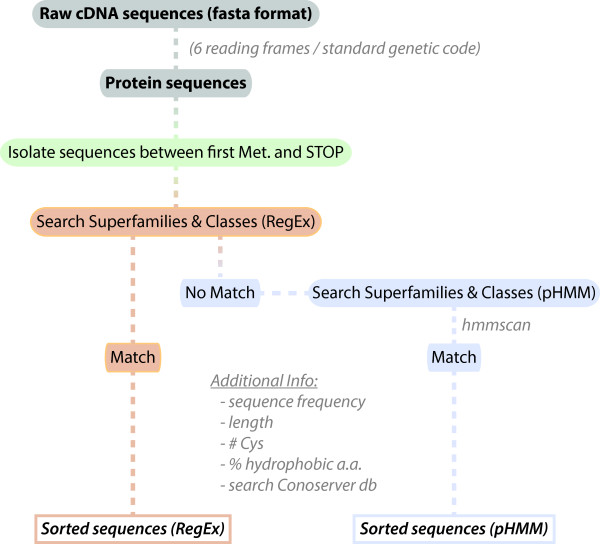
**Program pipeline.** The approach used in ConoSorter is divided into 4 steps: 1) translation of raw cDNA into amino acid sequences and formatting (input protein sequences can also be used directly with the corresponding command line argument); 2) independent searching for conopeptides superfamilies and classes using regular expressions; 3) matching pHMMs with unclassified sequence data set that didn’t provide hits with regular expressions; 4) calculation of additional sequence information (for details see “Methods” section).

The ConoSorter algorithm treats either cDNA or protein sequences according to a hierarchical step-wise process outlined in Figure 4. First, raw cDNA sequences are translated into all 6 reading frames. In order to obtain full-length precursor proteins, amino acid sequences delimited by a methionine and a stop codon are trimmed from the rest of the string and analysed. These sequences are submitted to a “rigid” Boolean search for signal / pro- / mature regions with high identity to known conopeptide superfamilies and classes using the regular expressions described above. The sequences are classified into (i) known superfamilies and/or classes, or (ii) sequences that did not return a match. For the first group, a score is given for each of the 3 regions independently - 1 if there is a match, 0 otherwise. Total scores for superfamilies and classes are then generated by simple addition of each region’s score. Instances in which there is conflict in the identification between distinct regions of the same sequence (i.e. the signal, pro- and/or mature regions have matches to different superfamilies) are also identified. The group of sequences that did not return matches to known superfamilies and classes is submitted to a more flexible stochastic search using the conopeptide-specific pHMMs described above and *hmmscan* script from the HMMER 3.0 package (*hmmscan*, like many other matching programs, use a default E-value threshold of 10). Total scores for superfamilies and classes are then calculated as the product of the E-values of the 3 independent regions.

Two separate sets of results are thus obtained, one for those with clear similarity to known superfamilies and classes and one for those that are potentially novel, which are stored in tabulated files called “Regex.tab” and “pHMM.tab”, respectively. We note that if a sequence has been assigned to a superfamily and/or a class based on the ER signal region, the amino acids before its specific signal motif are trimmed. The number of sequence(s) identical to a hit in the original input data set is reported, as well as the hit length, its cysteine content, and the percentage of hydrophobic residue in the N-terminal region of the sequence which is a hallmark for most newly synthesized proteins likely destined toward the secretory pathway. Finally, ConoSorter searches ConoServer database for previously described precursor conopeptide sequences.

### Analysis of the mRNA pool isolated from *Conus marmoreus* venom gland

The analysis of *Conus marmoreus* venom duct RNA sequencing data has recently been performed [18]. Briefly, mRNAs were sequenced with a Roche 454 pyrosequencer, and corresponding conopeptide sequences were identified by a BLAST homology search. To confirm the existence of new conopeptides, peptides isolated from *C. marmoreus* milked venom were sequenced by MS and matched to the conopeptide transcripts.

Here, we perform a reanalysis of this data using ConoSorter, as described above, with the addition of a number of computational steps to confidently identify novel conotoxin superfamilies and classes. Specifically, ConoSorter hits displaying matches only for the pro- and mature regions, as well as containing at least 60% hydrophobic amino acid in their N-terminal region were selected. This cutoff was chosen based on an analysis of all ConoServer conopeptide sequences with unique and complete signal regions - 644 in total, with a length and number of hydrophobic amino acids being 21.28 and 15.83 on average respectively (74.56% hydrophobicity with a standard deviation σ=6.59, and a minimum of 52.00%). These selected sequences were submitted to SignalP 4.0 in order to select sequences with a defined signal region [55]. Using these signal peptides, and those from annotated superfamilies, we then built a similarity matrix to ascertain the minimal intraspecific and maximal interspecific identity rates within and between known superfamilies (Additional file 5: Table S3). We submitted the isolated signal regions to the *CD-hit* clustering program by applying an identity cut-off of 75.00%. Signal sequences of the selected hits showing a similarity rate ≥75.00% were clustered. As an internal control we queried all empirical superfamilies and found that I2, L, P, M, I1, O1 families have an intraspecific conservation rate well below this threshold with 58.10%, 65.00%, 69.10%, 69.30%, 73.60% and 73.70% identity, respectively (Additional file 5: Table S3). Clusters displaying a maximum of 53.3% identity with any known superfamily were considered a putative novel superfamily. Signal regions of members of known and newly defined superfamilies were aligned using ClustalW in order to create a consensus identity cladogram. This analysis was also performed using MUSCLE algorithm, and showed a deviation of ±0.17% from the ClustalW results. Validation of novel conopeptides was performed using the previously published MS/MS data [18].

## Availability of supporting data

ConoSorter is licensed under the GNU General Public License version 3 (GPLv3) and freely available at http://sourceforge.net/p/conosorter.

## Abbreviations

ER: Endoplasmic reticulum; pHMM: profile hidden Markov model; MS: Mass spectrometry; RNA-seq: RNA sequencing; Bp: Base pair; ORF: Open reading frame.

## Competing interests

The authors declare that they have no competing interests.

## Authors’ contributions

VL carried out the design and conception of ConoSorter program, analysed and interpreted *Conus marmoreus* transcriptomic and proteomic data, conceived the figures and drafted the manuscript. SD participated in the design of ConoSorter program, has been involved in *Conus marmoreus* transcriptomic and proteomic data acquisition, and in revising the manuscript. AJ has performed *Conus marmoreus* proteomic data acquisition. RJL has provided *Conus marmoreus* transcriptomic sequencing data, and participated in revising the manuscript. RJT has participated in ConoSorter design, and has contributed in drafting the manuscript and revising it critically for important intellectual content. PFA has revised the manuscript and has given final approval of the version to be published. All authors read and approved the final manuscript.

## Supplementary Material

Additional file 1: Figure S1 Amino acid diversity in conopeptides. The position-specific inverse Simpson index, 1/λ, of amino acid for the ER signal (red), pro- (green), and mature (purple) conopeptide regions of the remaining gene superfamilie. For each amino acid position, a color gradient applies to the diversity index (from 0 in light to higher values in darker color). Click here for file

Additional file 2: Table S1 New isoforms of known *Conus marmoreus* precursor conopeptides. New isoforms of conopeptide precursors previously discovered in *Conus marmoreus* inferred from their known mature region (in blue). Peptide fragment coverage obtained by mass spectrometry analysis of the milked venom is represented in bold. The frequency of the sequence present in the mRNA pool, as well as the superfamily of the precursor conopeptide are also indicated in the table. Click here for file

Additional file 3: Figure S2 Isoforms of new precursor conopeptides classified into known superfamilies Click here for file

Additional file 4: Table S2 New precursor sequences found in *Conus marmoreus.* Their names, DDBJ accession numbers, and post-translational modifications of the peptide fragments (bold) generated by ProteinPilot 4.0 are mentioned in the above table. Click here for file

Additional file 5: Table S3 Similarity matrix of known and new conopeptide gene superfamilies. Known and new superfamilies are highlighted in red and grey respectively. Number between brackets following the superfamily name represents the conservation index of its members. Click here for file
