# Supplementary material for: Systematic interrogation of the Conus marmoreus venom duct transcriptome with ConoSorter reveals 158 novel conotoxins and 13 new gene superfamilies
Source: BMC Genomics. 2013 Oct 16;14:708. doi: 10.1186/1471-2164-14-708 (PMC3853152; doi:10.1186/1471-2164-14-708)
Supplement: Additional file 2: Table S1 — New isoforms of known Conus marmoreus precursor conopeptides. New isoforms of conopeptide precursors previously discovered in Conus marmoreus inferred from their known mature region (in blue). Peptide fragment coverage obtained by mass spectrometry analysis of the milked venom is represented in bold. The frequency of the sequence present in the mRNA pool, as well as the superfamily of the precursor conopeptide are also indicated in the table. [file 1471-2164-14-708-S2.docx]

Additional file 2: Table S1

| **Name** | **Freq.** | **Sequence** | **Superfamily** |
| --- | --- | --- | --- |
| New_Mr1.1_precursor | 2 | MSTVFLLVVLATTVVSFTSDRASDGRKAAAKDKASDLVALTVK**GCCSHPACSVNNPDIC**G | A |
| New_conomarphin_Mr1_precursor_1 | 1 | MSKLGVVLCIFLVLFPMATLQLDGDQTADHHADQRGQDLTEQQRNSKRVLKKRDWEY**HAHPKPNSFWT**LV | M |
| New_conomarphin_Mr1_precursor_2 | 2 | MSKLGVVLCIFLVLFPMATLQLDGDQTADRHADQRGQDLTEQHRNLKRVLKKRDWEY**HAHPKPNSFWT**LV | M |
| New_conomarphin_Mr1_precursor_3 | 1 | MSKLGVVLCIFLVLFPMATLQLDGDQTADRHADQRGQDLTEQQRNSKRVLKKRDWEY**HAHPKPNSFWT**LVERHQAGYSRTVVV | M |
| New_conomarphin_Mr2_precursor | 1 | MSKLGVVLCIFLVLFPMATLQLDGDQTADRHADQRGQDLTEQHQNLKRVLKKR**DWVNHAHPQPNSIWS**LV | M |
| New_contryphan_M_precursor_1 | 1 | MGKLTILVLVAAILLSTQVMVQDDRDQPA**DRNAVPRDDNPG**RARRKRMKVL**NESECPWHPWC**G | O2 |
| New_contryphan_M_precursor_2 | 1 | MGKLTILVLVAAVLLSTQAWFKGDRDQPADRNAVPRDDNPGRARRKRMK**VLNESECPWHPWC**G | O2 |
| New_cMrVIA_precursor | 1 | M**RCLPVLIILLLL**TASAPG**VVVLPKTEDDVPMSSVYGNGKSILRGILR**RVCCGYKLCHPC | T |
| New_CMrX_precursor_1 | 1 | M**RCLPVLIILLLL**TASAPGVDVLPKTEDDVPL**SSVYGNGKSIL**RGILR**KGICCGVSFCYPCL**TSMKGNDFG | T |
| New_CMrX_precursor_2 | 2 | M**RCLPVLIILLLL**TASAPGVDVLPKTEDDVSL**SSVYGNGKSIL**RGILRKGICCGVSFCYPC | T |
| New_CMrX_precursor_3 | 1 | M**RCLPVLIILLLL**TASAPGVDVLPKTEGDVPL**SSVYGNGKSIL**RGILRKGICCGVSFCYPC | T |
| New_MrIA_precursor_1 | 1 | M**RCLPVLIILLLLI**ASAPG**VVVLPKTEDDVPMSSVYGNGKSILRGILRNGVCCGYKLCHPC** | T |
| New_MrIA_precursor_2 | 3 | M**RCLPVLIILLLL**TASAAG**VVVLPKTEDDVPMSSVYGNGKSILRGILR**NGVCCGYKLCHPC | T |
| New_MrIA_precursor_3 | 2 | M**RCLPVLIILLLL**TASALG**VVVLPKTEDDVPMSSVYGNGKSILRGILR**NGVCCGYKLCHPC | T |
| New_MrIA_precursor_4 | 1 | M**RCLPVLIILLLL**TASAPD**VVVLPKTEDDVPMSSVYGNGKSILRGILR**NGVCCGYKLCHPC | T |
| New_MrIA_precursor_5 | 5 | M**RCLPVLIILLLL**TASAPGVDVLPKTEDDVPL**SSVYGNGKSIL**RGILRNGVCCGYKLCHPC | T |
| New_MrIA_precursor_6 | 1 | M**RCLPVLIILLLL**TASAPGVVVLPKTEDDVPL**SSVYGNGKSIL**RGILRNGVCCGYKLCHPC | T |

New isoforms of conopeptide precursors previously discovered in *Conus marmoreus* inferred from their known mature region (in blue). Peptide fragment coverage obtained by mass spectrometry analysis of the milked venom is represented in bold. The frequency of the sequence present in the mRNA pool, as well as the superfamily of the precursor conopeptide are also indicated in the table.
