# Supplementary material for: Systematic interrogation of the Conus marmoreus venom duct transcriptome with ConoSorter reveals 158 novel conotoxins and 13 new gene superfamilies
Source: BMC Genomics. 2013 Oct 16;14:708. doi: 10.1186/1471-2164-14-708 (PMC3853152; doi:10.1186/1471-2164-14-708)
Supplement: Additional file 4: Table S2 — New precursor sequences found in Conus marmoreus. Their names, DDBJ accession numbers, and post-translational modifications of the peptide fragments (bold) generated by ProteinPilot 4.0 are mentioned in the above table. [file 1471-2164-14-708-S4.docx]

Additional file 4: Table S2

| **Name** | **DDBJ Acc. #** | **Post-translational Modification(s)** |
| --- | --- | --- |
| Mr_precursor_001 | AB850695 |  |
| Mr_precursor_002 | AB850696 |  |
| Mr_precursor_003 | AB850697 |  |
| Mr_precursor_004 | AB850698 |  |
| Mr_precursor_005 | AB850699 |  |
| Mr_precursor_006 | AB850700 |  |
| Mr_precursor_007 | AB850701 |  |
| Mr_precursor_008 | AB850702 | Oxidation(H)@54. |
| Mr_precursor_009 | AB850703 |  |
| Mr_precursor_010 | AB850704 |  |
| Mr_precursor_011 | AB850705 | Oxidation(C)@59; Oxidation(H)@60; Oxidation(C)@63; Sulfo(Y)@64; Oxidation(C)@66. |
| Mr_precursor_012 | AB850706 |  |
| Mr_precursor_013 | AB850707 |  |
| Mr_precursor_014 | AB850708 |  |
| Mr_precursor_015 | AB850709 |  |
| Mr_precursor_016 | AB850710 |  |
| Mr_precursor_017 | AB850711 | Oxidation(C)@89; Oxidation(H)@90; Oxidation(C)@93; Sulfo(Y)@94; Oxidation(C)@96. |
| Mr_precursor_018 | AB850712 |  |
| Mr_precursor_019 | AB850713 |  |
| Mr_precursor_020 | AB850714 | Oxidation(D)@49; Oxidation(P)@52; Oxidation(P)@57; Oxidation(C)@60; Oxidation(C)@63; Oxidation(C)@64. |
| Mr_precursor_021 | AB850715 |  |
| Mr_precursor_022 | AB850716 | Deamidated(Q)@49; Deamidated(N)@53; Oxidation(C)@58; Oxidation(P)@59; Carboxy(E)@62; Oxidation(C)@63; Deamidated(Q)@66; Deamidated(Q)@68. |
| Mr_precursor_023 | AB850717 |  |
| Mr_precursor_024 | AB850718 | Deamidated(N)@27. |
| Mr_precursor_025 | AB850719 |  |
| Mr_precursor_026 | AB850720 |  |
| Mr_precursor_027 | AB850721 | Bromo(W)@76; Bromo(W)@80. |
| Mr_precursor_028 | AB850722 | Oxidation(P)@78. |
| Mr_precursor_029 | AB850723 |  |
| Mr_precursor_030 | AB850724 |  |
| Mr_precursor_031 | AB850725 |  |
| Mr_precursor_032 | AB850726 |  |
| Mr_precursor_033 | AB850727 |  |
| Mr_precursor_034 | AB850728 | Oxidation(C)@56; Deamidated(Q)@61; Oxidation(W)@66; Oxidation(P)@69; Oxidation(C)@73. |
| Mr_precursor_035 | AB850729 |  |
| Mr_precursor_036 | AB850730 |  |
| Mr_precursor_037 | AB850731 | Carboxy(E)@60. |
| Mr_precursor_038 | AB850732 |  |
| Mr_precursor_039 | AB850733 | Oxidation(M)@67. |
| Mr_precursor_040 | AB850734 | Deamidated(N)@65; Oxidation(C)@67; Oxidation(C)@68; Deamidated(R)@71; Deamidated(Q)@72; Oxidation(C)@73. |
| Mr_precursor_041 | AB850735 |  |
| Mr_precursor_042 | AB850736 |  |
| Mr_precursor_043 | AB850737 |  |
| Mr_precursor_044 | AB850738 |  |
| Mr_precursor_045 | AB850739 |  |
| Mr_precursor_046 | AB850740 |  |
| Mr_precursor_047 | AB850741 |  |
| Mr_precursor_048 | AB850742 |  |
| Mr_precursor_049 | AB850743 |  |
| Mr_precursor_050 | AB850744 |  |
| Mr_precursor_051 | AB850745 | Oxidation(C)@53; Oxidation(H)@54; Oxidation(W)@57. |
| Mr_precursor_052 | AB850746 |  |
| Mr_precursor_053 | AB850747 | Oxidation(H)@54. |
| Mr_precursor_054 | AB850748 | Oxidation(H)@54. |
| Mr_precursor_055 | AB850749 |  |
| Mr_precursor_056 | AB850750 |  |
| Mr_precursor_057 | AB850751 | Oxidation(C)@59; Oxidation(H)@60; Oxidation(C)@63; Sulfo(Y)@64; Oxidation(C)@66. |
| Mr_precursor_058 | AB850752 | Oxidation(H)@54. |
| Mr_precursor_059 | AB850753 | Oxidation(H)@54. |
| Mr_precursor_060 | AB850754 |  |
| Mr_precursor_061 | AB850755 |  |
| Mr_precursor_062 | AB850756 |  |
| Mr_precursor_063 | AB850757 |  |
| Mr_precursor_064 | AB850758 |  |
| Mr_precursor_065 | AB850759 |  |
| Mr_precursor_066 | AB850760 |  |
| Mr_precursor_067 | AB850761 |  |
| Mr_precursor_068 | AB850762 |  |
| Mr_precursor_069 | AB850763 |  |
| Mr_precursor_070 | AB850764 | Oxidation(C)@59; Oxidation(H)@60; Oxidation(C)@63; Sulfo(Y)@64; Oxidation(C)@66. |
| Mr_precursor_071 | AB850765 |  |
| Mr_precursor_072 | AB850766 |  |
| Mr_precursor_073 | AB850767 |  |
| Mr_precursor_074 | AB850768 |  |
| Mr_precursor_075 | AB850769 | Oxidation(H)@54. |
| Mr_precursor_076 | AB850770 | Oxidation(H)@54. |
| Mr_precursor_077 | AB850771 | Oxidation(H)@54. |
| Mr_precursor_078 | AB850772 | Oxidation(H)@53. |
| Mr_precursor_079 | AB850773 | Oxidation(H)@54. |
| Mr_precursor_080 | AB850774 | Oxidation(H)@54. |
| Mr_precursor_081 | AB850775 |  |
| Mr_precursor_082 | AB850776 | Oxidation(H)@56. |
| Mr_precursor_083 | AB850777 | Oxidation(H)@57. |
| Mr_precursor_084 | AB850778 | Oxidation(H)@60. |
| Mr_precursor_085 | AB850779 | Deamidated(R)@49; Oxidation(C)@51; Oxidation(C)@55; Bromo(H)@60; Oxidation(P)@61; Oxidation(C)@62. |
| Mr_precursor_086 | AB850780 | Oxidation(P)@52; Deamidated(N)@61; Oxidation(P)@62. |
| Mr_precursor_087 | AB850781 | Deamidated(R)@57; Oxidation(P)@62; Oxidation(C)@63; Oxidation(C)@64; Oxidation(W)@65. |
| Mr_precursor_088 | AB850782 | Oxidation(H)@60. |
| Mr_precursor_089 | AB850783 |  |
| Mr_precursor_090 | AB850784 |  |
| Mr_precursor_091 | AB850785 |  |
| Mr_precursor_092 | AB850786 |  |
| Mr_precursor_093 | AB850787 | Oxidation(M)@58; Deamidated(N)@63. |
| Mr_precursor_094 | AB850788 | Deamidated(N)@27. |
| Mr_precursor_095 | AB850789 |  |
| Mr_precursor_096 | AB850790 |  |
| Mr_precursor_097 | AB850791 | Deamidated(N)@27; Deamidated(N)@31. |
| Mr_precursor_098 | AB850792 |  |
| Mr_precursor_099 | AB850793 | Deamidated(N)@27. |
| Mr_precursor_100 | AB850794 |  |
| Mr_precursor_101 | AB850795 |  |
| Mr_precursor_102 | AB850796 |  |
| Mr_precursor_103 | AB850797 |  |
| Mr_precursor_104 | AB850798 |  |
| Mr_precursor_105 | AB850799 |  |
| Mr_precursor_106 | AB850800 |  |
| Mr_precursor_107 | AB850801 |  |
| Mr_precursor_108 | AB850802 |  |
| Mr_precursor_109 | AB850803 | Bromo(W)@76; Bromo(W)@80. |
| Mr_precursor_110 | AB850804 |  |
| Mr_precursor_111 | AB850805 |  |
| Mr_precursor_112 | AB850806 |  |
| Mr_precursor_113 | AB850807 |  |
| Mr_precursor_114 | AB850808 |  |
| Mr_precursor_115 | AB850809 |  |
| Mr_precursor_116 | AB850810 |  |
| Mr_precursor_117 | AB850811 |  |
| Mr_precursor_118 | AB850812 |  |
| Mr_precursor_119 | AB850813 |  |
| Mr_precursor_120 | AB850814 |  |
| Mr_precursor_121 | AB850815 |  |
| Mr_precursor_122 | AB850816 | Oxidation(C)@56; Deamidated(Q)@61; Oxidation(W)@66; Oxidation(P)@69; Oxidation(C)@73. |
| Mr_precursor_123 | AB850817 |  |
| Mr_precursor_124 | AB850818 |  |
| Mr_precursor_125 | AB850819 | Deamidated(Q)@56; Oxidation(M)@65. |
| Mr_precursor_126 | AB850820 | Deamidated(R)@46; Oxidation(C)@47; Oxidation(C)@48; Oxidation(H)@49; Bromo(W)@50; Oxidation(W)@50; Deamidated(N)@51; Oxidation(W)@52; Oxidation(C)@53; Oxidation(C)@59; Oxidation(C)@60. |
| Mr_precursor_127 | AB850821 |  |
| Mr_precursor_128 | AB850822 |  |
| Mr_precursor_129 | AB850823 | Deamidated(Q)@58; Deamidated(Q)@63; Deamidated(Q)@66; Oxidation(C)@68. |
| Mr_precursor_130 | AB850824 | Deamidated(Q)@58; Deamidated(Q)@63; Deamidated(Q)@66; Oxidation(C)@68. |
| Mr_precursor_131 | AB850825 |  |
| Mr_precursor_132 | AB850826 | Deamidated(N)@21; Deamidated(N)@25. |
| Mr_precursor_133 | AB850827 |  |
| Mr_precursor_134 | AB850828 |  |
| Mr_precursor_135 | AB850829 |  |
| Mr_precursor_136 | AB850830 |  |
| Mr_precursor_137 | AB850831 | Oxidation(P)@20; Deamidated(N)@22; Deamidated(N)@26; Oxidation(M)@53; Deamidated(N)@58. |
| Mr_precursor_138 | AB850832 | Oxidation(P)@20; Deamidated(N)@22; Deamidated(N)@26; Oxidation(M)@53; Deamidated(N)@58. |
| Mr_precursor_139 | AB850833 | Deamidated(N)@22. |
| Mr_precursor_140 | AB850834 | Deamidated(N)@22; Deamidated(N)@26. |
| Mr_precursor_141 | AB850835 |  |
| Mr_precursor_142 | AB850836 | Oxidation(P)@73. |
| Mr_precursor_143 | AB850837 |  |
| Mr_precursor_144 | AB850838 |  |
| Mr_precursor_145 | AB850839 |  |
| Mr_precursor_146 | AB850840 |  |
| Mr_precursor_147 | AB850841 |  |
| Mr_precursor_148 | AB850842 |  |
| Mr_precursor_149 | AB850843 |  |
| Mr_precursor_150 | AB850844 | Oxidation(P)@90. |
| Mr_precursor_151 | AB850845 |  |
| Mr_precursor_152 | AB850846 |  |
| Mr_precursor_153 | AB850847 |  |
| Mr_precursor_154 | AB850848 |  |
| Mr_precursor_155 | AB850849 | Sulfo(Y)@16. |
| Mr_precursor_156 | AB850850 | Sulfo(Y)@16. |
| Mr_precursor_157 | AB850851 |  |
| Mr_precursor_158 | AB850852 |  |
| New_Mr1.1_precursor | AB850863 |  |
| New_conomarphin_Mr1_precursor_1 | AB850857 | Oxidation(H)@58; Oxidation(H)@60; Oxidation(P)@61; Oxidation(P)@63; Oxidation(N)@64; Deamidated(N)@64; Oxidation(W)@67. |
| New_conomarphin_Mr1_precursor_2 | AB850858 | Oxidation(H)@58; Oxidation(H)@60; Oxidation(P)@61; Oxidation(P)@63; Oxidation(N)@64; Deamidated(N)@64; Oxidation(W)@67. |
| New_conomarphin_Mr1_precursor_3 | AB850859 | Oxidation(H)@58; Oxidation(H)@60; Oxidation(P)@61; Oxidation(P)@63; Oxidation(N)@64; Deamidated(N)@64; Oxidation(W)@67. |
| New_conomarphin_Mr2_precursor | AB850860 | Deamidated(N)@57; Oxidation(P)@61; Oxidation(P)@63; Deamidated(N)@64. |
| New_contryphan_M_precursor_1 | AB850861 |  |
| New_contryphan_M_precursor_2 | AB850862 | Deamidated(N)@52. |
| New_cMrVIA_precursor | AB850853 |  |
| New_CMrX_precursor_1 | AB850854 | Oxidation(P)@5; Oxidation(P)@60; Oxidation(C)@61. |
| New_CMrX_precursor_2 | AB850855 |  |
| New_CMrX_precursor_3 | AB850856 |  |
| New_MrIA_precursor_1 | AB850864 | Oxidation(P)@5; Oxidation(P)@31; Oxidation(M)@32; Deamidated(N)@38; Deamidated(N)@49; Oxidation(C)@52; Oxidation(C)@58; Oxidation(P)@60; Oxidation(C)@61. |
| New_MrIA_precursor_2 | AB850865 |  |
| New_MrIA_precursor_3 | AB850866 |  |
| New_MrIA_precursor_4 | AB850867 |  |
| New_MrIA_precursor_5 | AB850868 |  |
| New_MrIA_precursor_6 | AB850869 |  |

New precursor sequences found in *Conus marmoreus.* Their names, DNA Data Bank of Japan (DDBJ) accession numbers, and post-translational modifications of the peptide fragments (bold) generated by ProteinPilot4 are mentioned in the above table.
