# Supplementary material for: Systematic interrogation of the Conus marmoreus venom duct transcriptome with ConoSorter reveals 158 novel conotoxins and 13 new gene superfamilies
Source: BMC Genomics. 2013 Oct 16;14:708. doi: 10.1186/1471-2164-14-708 (PMC3853152; doi:10.1186/1471-2164-14-708)
Supplement: Additional file 5: Table S3 — Similarity matrix of known and new conopeptide gene superfamilies. Known and new superfamilies are highlighted in red and grey respectively. Number between brackets following the superfamily name represents the conservation index of its members. [file 1471-2164-14-708-S5.pdf]

	A	B1	B2	B3	C	D	E	F	G	H	I1	I2	I3	J	K	L	M	N	O1	O2	O3	P	S	T	V	Y	H2	I4	M2	N2	O4	Q	R	U	W	X	Y2	Y3	Z
A (0.905)		0.139	0.188	0.233	0.134	0.160	0.280	0.252	0.316	0.357	0.224	0.136	0.170	0.216	0.520	0.309	0.091	0.312	0.133	0.164	0.325	0.157	0.322	0.231	0.257	0.250	0.278	0.200	0.133	0.148	0.263	0.167	0.200	0.088	0.185	0.222	0.296	0.160	0.115
B1 (0.904)	0.139		0.235	0.208	0.196	0.144	0.192	0.229	0.302	0.216	0.218	0.061	0.229	0.080	0.273	0.154	0.237	0.233	0.168	0.255	0.117	0.114	0.188	0.112	0.207	0.350	0.211	0.202	0.157	0.252	0.182	0.144	0.162	0.178	0.154	0.309	0.308	0.242	0.162
B2 (*)	0.188	0.235		0.313	0.193	0.111	0.188	0.250	0.222	0.318	0.199	0.237	0.190	0.250	0.353	0.011	0.201	0.188	0.257	0.385	0.169	0.268	0.128	0.465	0.253	0.188	0.250	0.063	0.302	0.118	0.176	0.160	0.158	0.125	0.176	0.250	0.190	0.368	0.077
B3 (*)	0.233	0.208	0.313		0.218	0.214	0.200	0.208	0.179	0.286	0.222	0.171	0.202	0.200	0.174	0.133	0.135	0.250	0.089	0.235	0.216	0.187	0.134	0.196	0.159	0.333	0.313	0.107	0.258	0.174	0.045	0.207	0.185	0.118	0.160	0.240	0.250	0.080	0.143
C (0.894)	0.134	0.196	0.193	0.218		0.129	0.273	0.140	0.152	0.212	0.103	0.061	0.233	0.095	0.186	0.192	0.166	0.170	0.107	0.143	0.129	0.082	0.145	0.145	0.147	0.275	0.068	0.052	0.184	0.091	0.136	0.146	0.186	0.075	0.134	0.140	0.221	0.004	0.204
D (0.823)	0.160	0.144	0.111	0.214	0.129		0.130	0.292	0.421	0.348	0.407	0.148	0.252	0.148	0.217	0.309	0.263	0.520	0.251	0.352	0.360	0.264	0.265	0.366	0.270	0.478	0.300	0.200	0.182	0.217	0.089	0.115	0.208	0.097	0.250	0.087	0.333	0.200	0.120
E (*)	0.280	0.192	0.188	0.200	0.273	0.130		0.286	0.211	0.150	0.140	0.233	0.169	0.136	0.278	0.172	0.209	0.091	0.237	0.263	0.169	0.134	0.255	0.172	0.252	0.200	0.063	0.292	0.219	0.136	0.252	0.160	0.056	0.278	0.130	0.333	0.238	0.136	0.045
F (*)	0.252	0.229	0.250	0.208	0.140	0.292	0.286		0.333	0.318	0.182	0.112	0.306	0.095	0.190	0.236	0.305	0.200	0.140	0.300	0.242	0.233	0.231	0.231	0.245	0.500	0.222	0.240	0.258	0.190	0.294	0.222	0.273	0.179	0.136	0.136	0.304	0.308	0.200
G (*)	0.316	0.302	0.222	0.179	0.152	0.421	0.211	0.333		0.350	0.159	0.129	0.202	0.259	0.263	0.355	0.388	0.474	0.167	0.318	0.423	0.288	0.321	0.320	0.270	0.526	0.278	0.316	0.223	0.348	0.176	0.185	0.130	0.105	0.091	0.053	0.300	0.227	0.273
H (0.974)	0.357	0.216	0.318	0.286	0.212	0.348	0.150	0.318	0.350		0.192	0.227	0.152	0.200	0.227	0.152	0.275	0.417	0.149	0.294	0.294	0.301	0.242	0.186	0.279	0.381	0.400	0.320	0.145	0.190	0.048	0.269	0.192	0.087	0.318	0.217	0.360	0.261	0.208
I1 (0.736)	0.224	0.218	0.199	0.222	0.103	0.407	0.140	0.182	0.159	0.192		0.229	0.364	0.249	0.192	0.318	0.142	0.259	0.223	0.142	0.251	0.144	0.194	0.238	0.316	0.311	0.165	0.227	0.273	0.227	0.194	0.163	0.138	0.093	0.216	0.143	0.176	0.169	0.163
I2 (0.581)	0.136	0.061	0.237	0.171	0.061	0.148	0.233	0.112	0.129	0.227	0.229		0.102	0.175	0.138	0.056	0.182	0.142	0.138	0.180	0.142	0.209	0.237	0.229	0.157	0.200	0.182	0.382	0.144	0.184	0.150	0.102	0.148	0.098	0.180	0.107	0.220	0.182	0.202
I3 (0.929)	0.170	0.229	0.190	0.202	0.233	0.252	0.169	0.306	0.202	0.152	0.364	0.102		0.219	0.152	0.361	0.275	0.229	0.346	0.205	0.138	0.340	0.245	0.355	0.165	0.371	0.136	0.145	0.188	0.350	0.242	0.162	0.219	0.102	0.002	0.202	0.219	0.138	0.252
J (0.905)	0.216	0.080	0.250	0.200	0.095	0.148	0.136	0.095	0.259	0.200	0.249	0.175	0.219		0.087	0.104	0.091	0.148	0.128	0.263	0.162	0.082	0.045	0.147	0.198	0.286	0.200	0.222	0.182	0.160	0.127	0.214	0.083	0.074	0.250	0.182	0.227	0.222	0.133
K (1.000)	0.520	0.273	0.353	0.174	0.186	0.217	0.278	0.190	0.263	0.227	0.192	0.138	0.152	0.087		0.159	0.131	0.273	0.182	0.263	0.377	0.136	0.231	0.186	0.197	0.200	0.125	0.091	0.091	0.091	0.138	0.107	0.217	0.190	0.087	0.200	0.208	0.083	0.154
L (0.650)	0.309	0.154	0.011	0.133	0.192	0.309	0.172	0.236	0.355	0.152	0.318	0.056	0.361	0.104	0.159		0.162	0.259	0.405	0.211	0.294	0.114	0.264	0.283	0.172	0.209	0.068	0.159	0.218	0.199	0.294	0.044	0.199	0.257	0.112	0.159	0.225	0.207	0.207
M (0.693)	0.091	0.237	0.201	0.135	0.166	0.263	0.209	0.305	0.388	0.275	0.142	0.182	0.275	0.091	0.131	0.162		0.307	0.222	0.237	0.256	0.202	0.405	0.225	0.293	0.388	0.249	0.173	0.477	0.198	0.192	0.129	0.098	0.076	0.126	0.105	0.215	0.215	0.199
N (0.972)	0.312	0.233	0.188	0.250	0.170	0.520	0.091	0.200	0.474	0.417	0.259	0.142	0.229	0.148	0.273	0.259	0.307		0.166	0.254	0.522	0.326	0.284	0.277	**0.533**	0.500	0.313	0.160	0.223	0.391	0.263	0.143	0.125	0.097	0.167	0.048	0.375	0.192	0.115
O1 (0.737)	0.133	0.168	0.257	0.089	0.107	0.251	0.237	0.140	0.167	0.149	0.223	0.138	0.346	0.128	0.182	0.405	0.222	0.166		0.275	0.203	0.213	0.169	0.198	0.251	0.281	0.297	0.166	0.277	0.186	0.492	0.173	0.091	0.135	0.099	0.127	0.220	0.053	0.128
O2 (0.788)	0.164	0.255	0.385	0.235	0.143	0.352	0.263	0.300	0.318	0.294	0.142	0.180	0.205	0.263	0.263	0.211	0.237	0.254	0.275		0.245	0.194	0.143	0.143	0.180	0.374	0.190	0.204	0.099	0.134	0.180	0.146	0.295	0.182	0.089	0.138	0.295	0.124	0.204
O3 (0.879)	0.325	0.117	0.169	0.216	0.129	0.360	0.169	0.242	0.423	0.294	0.251	0.142	0.138	0.162	0.377	0.294	0.256	0.522	0.203	0.245		0.240	0.368	0.256	0.325	0.352	0.169	0.242	0.218	0.145	0.087	0.156	0.042	0.130	0.127	0.095	0.252	0.202	0.229
P (0.691)	0.157	0.114	0.268	0.187	0.082	0.264	0.134	0.233	0.288	0.301	0.144	0.209	0.340	0.082	0.136	0.114	0.202	0.326	0.213	0.194	0.240		0.340	0.266	0.236	0.370	0.259	0.231	0.261	0.209	0.268	0.111	0.240	0.121	0.303	0.214	0.312	0.249	0.187
S (0.913)	0.322	0.188	0.128	0.134	0.145	0.265	0.255	0.231	0.321	0.242	0.194	0.237	0.245	0.045	0.231	0.264	0.405	0.284	0.169	0.143	0.368	0.340		0.294	0.238	0.305	0.190	0.212	0.394	0.322	0.233	0.087	0.212	0.140	0.134	0.195	0.337	0.140	0.277
T (0.771)	0.231	0.112	0.465	0.196	0.145	0.366	0.172	0.231	0.320	0.186	0.238	0.229	0.355	0.147	0.186	0.283	0.225	0.277	0.198	0.143	0.256	0.266	0.294		0.311	0.320	0.213	0.277	0.192	0.308	0.180	0.119	0.134	0.132	0.095	0.147	0.254	0.204	0.124
V (0.842)	0.257	0.207	0.253	0.159	0.147	0.270	0.252	0.245	0.270	0.279	0.316	0.157	0.165	0.198	0.197	0.172	0.293	**0.533**	0.251	0.180	0.325	0.236	0.238	0.311		0.218	0.315	0.112	0.184	0.218	0.167	0.131	0.131	0.157	0.223	0.107	0.256	0.256	0.233
Y (*)	0.250	0.350	0.188	0.333	0.275	0.478	0.200	0.500	0.526	0.381	0.311	0.200	0.371	0.286	0.200	0.209	0.388	0.500	0.281	0.374	0.352	0.370	0.305	0.320	0.218		0.250	0.250	0.199	0.286	0.176	0.160	0.273	0.150	0.095	0.050	0.400	0.409	0.240
H2 (*)	0.278	0.211	0.250	0.313	0.068	0.300	0.063	0.222	0.278	0.400	0.165	0.182	0.136	0.200	0.125	0.068	0.249	0.313	0.297	0.190	0.169	0.259	0.190	0.213	0.315	0.250		0.188	0.217	0.267	0.125	0.190	0.143	0.059	0.176	0.158	0.263	0.278	0.333
I4 (1.000)	0.200	0.202	0.063	0.107	0.052	0.200	0.292	0.240	0.316	0.320	0.227	0.382	0.145	0.222	0.091	0.159	0.173	0.160	0.166	0.204	0.242	0.231	0.212	0.277	0.112	0.250	0.188		0.133	0.240	0.202	0.143	0.115	0.097	0.200	0.136	0.320	0.240	0.080
M2 (0.826)	0.133	0.157	0.302	0.258	0.184	0.182	0.219	0.258	0.223	0.145	0.273	0.144	0.188	0.182	0.091	0.218	0.477	0.223	0.277	0.099	0.218	0.261	0.394	0.192	0.184	0.199	0.217	0.133		0.182	0.101	0.138	0.223	0.149	0.174	0.152	0.127	0.127	0.219
N2 (*)	0.148	0.252	0.118	0.174	0.091	0.217	0.136	0.190	0.348	0.190	0.227	0.184	0.350	0.160	0.091	0.199	0.198	0.391	0.186	0.134	0.145	0.209	0.322	0.308	0.218	0.286	0.267	0.240	0.182		0.176	0.174	0.130	0.097	0.091	0.217	0.261	0.280	0.208
O4 (0.903)	0.263	0.182	0.176	0.045	0.136	0.089	0.252	0.294	0.176	0.048	0.194	0.150	0.242	0.127	0.138	0.294	0.192	0.263	0.492	0.180	0.087	0.268	0.233	0.180	0.167	0.176	0.125	0.202	0.101	0.176		0.169	0.169	0.295	0.114	0.102	0.176	0.233	0.169
Q (*)	0.167	0.144	0.160	0.207	0.146	0.115	0.160	0.222	0.185	0.269	0.163	0.102	0.162	0.214	0.107	0.044	0.129	0.143	0.173	0.146	0.156	0.111	0.087	0.119	0.131	0.160	0.190	0.143	0.138	0.174	0.169		0.250	0.227	0.250	0.238	0.179	0.179	0.154
R (*)	0.200	0.162	0.158	0.185	0.186	0.208	0.056	0.273	0.130	0.192	0.138	0.148	0.219	0.083	0.217	0.199	0.098	0.125	0.091	0.295	0.042	0.240	0.212	0.134	0.131	0.273	0.143	0.115	0.223	0.130	0.169	0.250		0.071	0.217	0.250	0.167	0.240	0.115
U (*)	0.088	0.178	0.125	0.118	0.075	0.097	0.278	0.179	0.105	0.087	0.093	0.098	0.102	0.074	0.190	0.257	0.076	0.097	0.135	0.182	0.130	0.121	0.140	0.132	0.157	0.150	0.059	0.097	0.149	0.097	0.295	0.227	0.071		0.130	0.071	0.083	0.080	0.207
W (1.000)	0.185	0.154	0.176	0.160	0.134	0.250	0.130	0.136	0.091	0.318	0.216	0.180	0.002	0.250	0.087	0.112	0.126	0.167	0.099	0.089	0.127	0.303	0.134	0.095	0.223	0.095	0.176	0.200	0.174	0.091	0.114	0.250	0.217	0.130		0.200	0.280	0.200	0.154
X (1.000)	0.222	0.309	0.250	0.240	0.140	0.087	0.333	0.136	0.053	0.217	0.143	0.107	0.202	0.182	0.200	0.159	0.105	0.048	0.127	0.138	0.095	0.214	0.195	0.147	0.107	0.050	0.158	0.136	0.152	0.217	0.102	0.238	0.250	0.071	0.200		0.217	0.280	0.182
Y2 (1.000)	0.296	0.308	0.190	0.250	0.221	0.333	0.238	0.304	0.300	0.360	0.176	0.220	0.219	0.227	0.208	0.225	0.215	0.375	0.220	0.295	0.252	0.312	0.337	0.254	0.256	0.400	0.263	0.320	0.127	0.261	0.176	0.179	0.167	0.083	0.280	0.217		0.240	0.217
Y3 (*)	0.160	0.242	0.368	0.080	0.004	0.200	0.136	0.308	0.227	0.261	0.169	0.182	0.138	0.222	0.083	0.207	0.215	0.192	0.053	0.124	0.202	0.249	0.140	0.204	0.256	0.409	0.278	0.240	0.127	0.280	0.233	0.179	0.240	0.080	0.200	0.280	0.240		0.080
Z (*)	0.115	0.162	0.077	0.143	0.204	0.120	0.045	0.200	0.273	0.208	0.163	0.202	0.252	0.133	0.154	0.207	0.199	0.115	0.128	0.204	0.229	0.187	0.277	0.124	0.233	0.240	0.333	0.080	0.219	0.208	0.169	0.154	0.115	0.207	0.154	0.182	0.217	0.080	
